# Hyperspectral near infrared imaging quantifies the heterogeneity of carbon materials

**DOI:** 10.1038/s41598-018-28889-7

**Published:** 2018-07-11

**Authors:** Mikko Mäkelä, Paul Geladi

**Affiliations:** 1 Aalto University, Department of Bioproducts and Biosystems, PO Box 16300, 00076 Aalto, Finland; 2 Swedish University of Agricultural Sciences, Department of Forest Biomaterials and Technology, Skogsmarksgränd, 90183 Umeå, Sweden

## Abstract

For many applications heterogeneity is a direct indicator of material quality. Reliable determination of chemical heterogeneity is however not a trivial task. Spectral imaging can be used for determining the spatial distribution of an analyte in a sample, thus transforming each pixel of an image into a sampling cell. With a large amount of image pixels, the results can be evaluated using large population statistics. This enables robust determination of heterogeneity in biological samples. We show that hyperspectral imaging in the near infrared (NIR) region can be used to reliably determine the heterogeneity of renewable carbon materials, which are promising replacements for current fossil alternatives in energy and environmental applications. This method allows quantifying the variation in renewable carbon and other biological materials that absorb in the NIR region. Reliable determination of heterogeneity is also a valuable tool for a wide range of other chemical imaging applications.

## Introduction

For many applications variation or heterogeneity is a direct indicator of material quality. Reliable estimation of chemical heterogeneity is however not a trivial task. The material needs to be correctly sampled to describe its true time or space dependent variation, which requires obtaining and analysing a sufficient number of samples while minimizing the uncertainty of the analytical procedure. Although an entire theory of representative sampling has been developed for this purpose^1,2^, fast and reliable quantification of heterogeneity from individual samples or real-time monitoring of entire material streams remains challenging.

Spectral imaging can combine e.g. vibrational spectroscopy with the spatial attributes of an image. Mathematical operations can thus be performed in both the spectral and spatial domains, which enables relating chemical information of different analytes with individual image pixels. As an example, vibrational modes of different molecular bonds can be measured within an entire image area through absorbance in the near infrared (NIR) or infrared (IR) region. As opposed to fundamental vibrational modes detected with IR, NIR wavelengths provide information on the overtones and combination bands of hydrogen and other bonds. Molecular absorptivity in the NIR region is thus orders of magnitude weaker than in the IR, which requires longer path lengths for obtaining undistorted spectra, but provides an advantage in sample preparation and in analysing larger heterogeneous samples^3,4^. Spectral interpretation in the NIR is however more complicated and is often performed through calibration based on a known reference method. The term hyperspectral imaging is generally used when spectral images are continuously recorded on tens or even several hundred different wavelengths^5^.

After a calibration model has been determined based on the known concentrations of calibration samples, the properties of individual image pixels can be predicted. In addition to the average concentration of an analyte in a sample, spectral imaging thus enables determining its spatial distribution^6^. Each pixel then becomes a sampling cell. With images that contain a large number of sample pixels, the results can be evaluated using large population statistics^7^. This enables developing a reliable variation metric for describing material heterogeneity.

Although spectral imaging has its roots in remote sensing^3,6,8^, it has been finding its way also to the chemical and materials science communities. As an example, in-line hyperspectral NIR imaging has recently been used for studying the spatial properties of adhesive layers in textile laminates^9^. NIR imaging below the diffraction limit has recently been reported for studying the behavior of DNA walkers^10^. NIR and Raman imaging have also been used for identification of counterfeit drugs^11,12^. Wilczyński *et al*.^12^ used the analysis of gray value intensities for quantitative evaluation of heterogeneity in counterfeit tablets. No calibration model was determined and differences in heterogeneity were based on spatial and spectral information alone. Recently de Moura França *et al*.^13^ also illustrated the use of homogeneity curves for analyzing heterogeneity of binary images.

Here, we demonstrate for the first time the use of hyperspectral NIR imaging for quantifying the heterogeneity of renewable carbon materials after multivariate image regression. Biomass-derived carbon is a promising material as it can be prepared from renewable feedstocks and used for replacing current fossil alternatives in a range of energy and environmental applications^14^. The chemical and physical properties of carbon materials can be fine-tuned for a specific application through the use of additives or subsequent activation steps^15,16^. Independent of the choice of method or final application, variation in the properties of the carbon material affect the quality of the final product. In the future, hyperspectral NIR imaging and heterogeneity estimates can be used for monitoring the quality of renewable carbon and other biological materials that absorb in NIR region.

## Methods

### Carbon materials

Carbon materials were hydrothermally prepared from chemical sludge produced at a wastewater treatment plant of a pulp and paper mill. An experimental design was used to systematically produce carbon materials with different properties. In the design carbonization temperature (180–260 °C) and the moisture content of sludge (10.2–4.4 kg H_2_O kg^−1^ db) were varied on three different levels. A total of 11 experiments were performed including three repeated center-points (Table 1). The hydrothermal experiments were performed in a 280 mL autoclave (Büchi Limbo, Büchi AG) using 200 g of wet sludge. The reactor was purged with 2 MPa nitrogen and heated to treatment temperature under autogenous pressure followed by an isothermal holding time of 1 h. Once the reactor was cooled to room temperature, the non-condensable gases were discarded and the slurry was vacuum-filtered using a 25 µm pore size Whatman filter paper. The filtered carbon material was dried at 105 °C overnight. The ash and carbon contents of the dried samples were determined according to respective European standards SS-EN 14775 and SS-EN 15407. The final carbon content was given as a percentage of dry, ash-free (daf) char. After the analyses, the samples were sieved through a 250 µm sieve (Retsch GmbH), which provided a total of 33 samples in three different size fractions including the original unsieved material.

**Table 1:** The hydrothermal experiments.

Experiment	Carbonization temperature (°C)	Moisture content (kg H_2_O kg^−1^ db)	Dry solids (%)
1	180	10	8.9
2	260	10	8.9
3	180	4.4	19
4	260	4.4	19
5	180	7.3	12
6	260	7.3	12
7	220	10	8.9
8	220	4.4	19
9	220	7.3	12
10	220	7.3	12
11	220	7.3	12

db = dry basis.

### Spectral imaging

Hyperspectral imaging was performed with a Specim (Specim, Spectral Imaging, Ltd.) camera equipped with an OLES30 lens provided by Specim. Three images were taken, one for each size fraction. Two rows of quartz halogen lamps generated polychromatic light and the reflected wavelengths were separated by a grating-prism monochromator followed by a HgCdTe detector array. The spectral range was limited to 950–2550 nm with a spectral resolution of approximately 6 nm. The samples were placed in black spherical polypropylene screw caps with a diameter of 23 mm and a depth of 8 mm. The camera was operated in line-scanning mode where a line of 388 pixels was continuously recorded on 288 wavelengths. The samples moved under the camera on a moving belt generating an image. The number of lines and samples on the moving belt thus determined image size. The acquisition time was 10 ms per line, resulting in approximately 14 s per image. The field of view was set to 50 mm, which resulted in a pixel size of 0.13 × 0.13 mm. The absorbance in each pixel was calculated based on measured Spectralon white reference and dark current intensities.

### Image and data analysis

Backgrounds, sample holders and potential dead pixels were removed from the individual images using principal component analysis (PCA)^17^. The hyperspectral image of each unsieved sample was split into four individual 90 degree segments, which resulted in a total of 44 calibration samples on 276 wavelengths. The respective median spectra were calculated and further split into 33 calibration and 11 validation objects. A calibration model was then determined based on partial least squares (PLS) regression^18^ on determined reference values. Spectral preprocessing was performed through the standard normal variate (SNV) transformation and mean centering to minimize the effects of light scattering. The reference carbon values were mean centered. The root mean squared errors of prediction (RMSEP) based on the validation set were calculated as:

1 $$RMSEP=\sqrt{\frac{\sum _{i=1}^{n}{({y}_{i}-{\hat{y}}_{i})}^{2}}{n}}$$

where *y*_*i*_ and ${\hat{y}}_{i}$ denote the measured and predicted values, respectively, and *n* the number of predictions. In addition, prediction bias was calculated as:

2 $$Bias=\frac{\sum _{i=1}^{n}({y}_{i}-{\hat{y}}_{i})}{n}$$

RMSEP was used to describe model range error ratio (RER) and residual prediction deviation (RPD) parameters through:

3 $$RER=\frac{({y}_{max}-{y}_{min})}{RMSEP}$$

4 $$RPD=\sqrt{\frac{\sum _{i=1}^{n}{({y}_{i}-{\bar{y}}_{i})}^{2}}{n-1}}RMSE{P}^{-1}$$

Data analysis and plotting were performed with the Evince (Prediktera AB), Matlab (The Mathworks, Inc.) PLS Toolbox (Eigenvector Research, Inc.) and OriginPro (Originlab Corp.) software packages. Data availability; the datasets analysed during this study are available from the corresponding author on reasonable request.

## Results and Discussion

**Figure 1:**
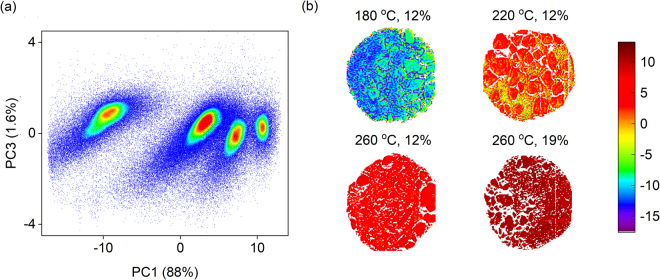
(**a**) Principal components scores of the cleaned sample image and (**b**) a score image of four samples based on the first principal component which explained 88% of data variation. Samples labels in (**b**) show carbonization conditions given in Table 1.

Backgrounds and unnecessary pixels were removed from the images with the help of PCA. The first three principal components explained 96–97% of variation in the cleaned images. The first component described the mean spectra, while the second and third components included more detailed spectral features. Based on the score images, the first component described differences between the different samples as the second and third mainly included variation within the individual samples due to e.g. uneven illumination of the sample surfaces. PCA scores of the first and third component showed that the carbon samples contained four different groups, which were mainly separated by carbonization temperature, Fig. 1a. The samples carbonized at 260 °C were further separated into two groups. The effect of carbonization temperature was also clearly visible in the score images (Fig. 1b). This indicated that their chemical differences were visible in the NIR spectra. Once the images had been cleaned, the samples were split into four different parts and the respective median spectra were used for calibration against the reference carbon contents of the samples. The first wavelengths within 950–1000 nm were discarded as they mainly consisted of noise.

**Figure 2:**
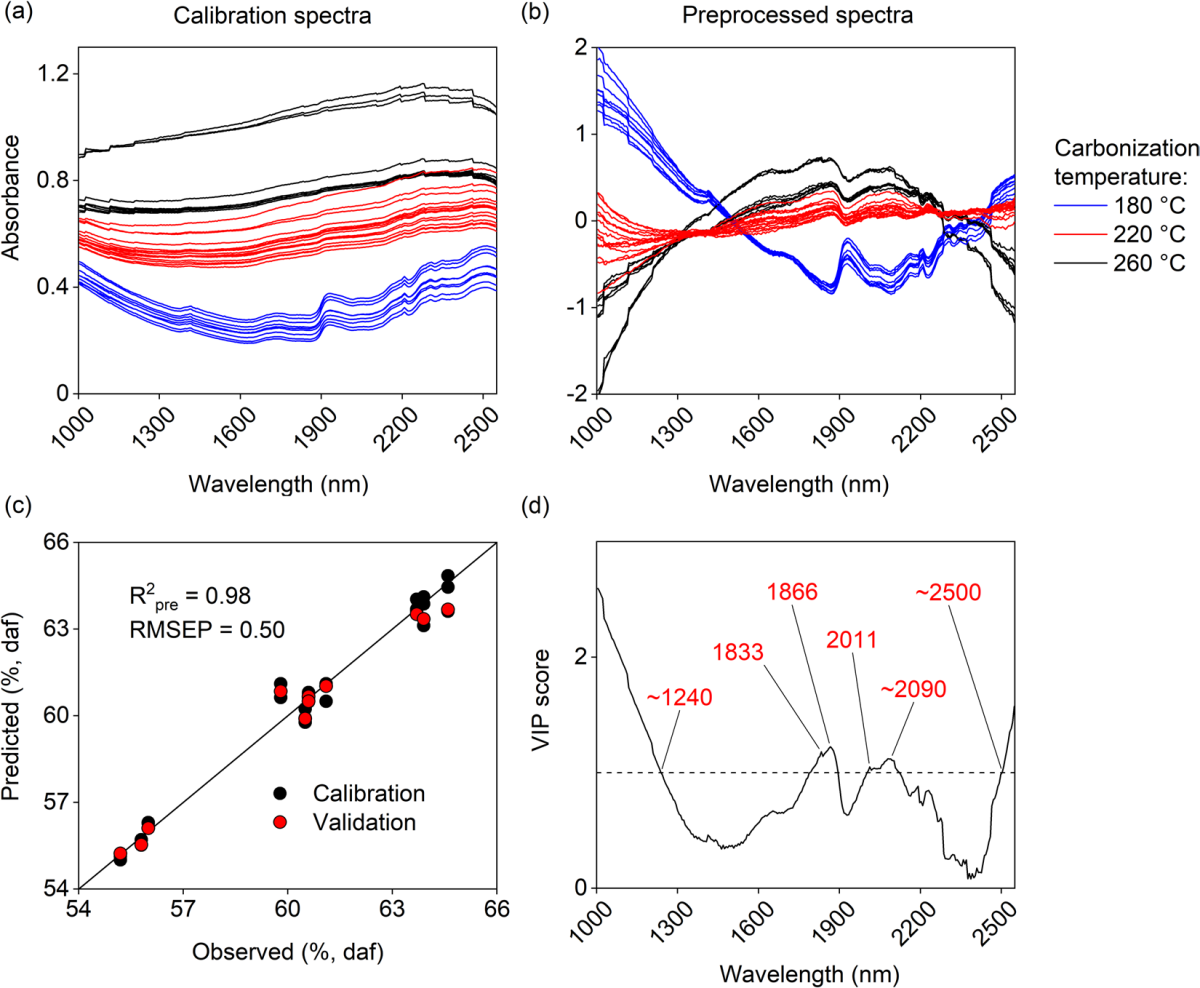
(**a**) Original and (**b**) preprocessed calibration spectra. Preprocessing was based on SNV transformation and mean centering. (**c**) Predicted vs. observed carbon contents based on the final PLS model, where the 45° line illustrates a perfect fit. (**d**) VIP scores of the PLS model.

The calibration spectra before preprocessing showed increased loss of spectral features with increasing carbonization temperature, Fig. 2a. Especially the peaks at approximately 1410, 1740, 1930 and 2510 nm that correspond with the stretching of O-H, S-H, C-O and C-H from C-H and CH_2_ groups^19,20^ completely disappeared from the samples produced at higher temperatures. A calibration model was however successfully determined based on preprocessed spectra (Fig. 2b). The final PLS model was composed of 4 latent variables and explained 98% of variation in the calibration and validation data (Fig. 2c). The determined RMSEP were 0.50% carbon (daf) combined with low prediction bias (−0.13). The model RER and ratio RPD parameters related the prediction errors to the original distribution of reference values and were 19 and 6.6, respectively. This indicated that the model performed well and was suitable for quality control applications^3^.

PLS VIP scores based on the model suggested that wavelengths below 1240, within 1830–1870 and 2010–2090, and above 2500 nm were especially important for the predictive ability of the model (Fig. 2d). Wavelengths below 1240 nm generally contain the second overtones of C-H strains of CH and CH_2_ groups and the CH_3_ groups of aromatic moieties^19^. The preprocessed calibration spectra showed significant variation between the different sample groups in this range (Fig. 2b), which was also indicated by the loading vector of the first latent variable explaining 94% of the variation in the reference values. Shorter NIR wavelengths are also more strongly affected by light scattering and sample color. The effects of scattering are however complicated due to the presence of absorbing constituents and variation in physical properties such as particle size and shape, surface properties and sample packing^21,22^. Increasing carbonization temperature generally leads to a higher carbon content and a darker sample color, which can manifest as increased absorbance on shorter wavelengths^23^. The O-H and C-O strains and the second overtone of C-O strain can normally be detected at 1830 nm^20^. The second overtone of the C=O strain and the O-H deformation and strain generally absorb within 2030–2080 nm^19^. Absorbance of C-O strains and C-H strains from C-H and CH_2_ groups have been reported at 2506–2519 nm^19^. Based on additional chemical analyses, the O/C ratio of the feed material decreased from 0.61 (daf) to 0.29–0.46 (daf) during the experiments, which explains changes in the absorbance of C-O and C=O bonds. The respective H/C ratio decreased slightly from 1.7 (daf) to 1.4–1.6 (daf) suggesting changes in C-H and possibly O-H bonds likely through the decomposition of carbohydrates. It should be noted that the spectral resolution of hyperspectral cameras is generally not as high as those of spectrometers, which can generate a level of uncertainty to the interpretation of spectral assignments. The PLS model however enabled reliable calibration as indicated by the determined performance parameters.

Once the suitable calibration model was obtained, the carbon contents of individual image pixels were predicted. Two more images were obtained after sieving the original samples through a 250 μm sieve. The sieving enabled testing the hypothesis that variation in the different size fractions could be determined based on the predicted pixel data. The pixel populations of individual samples were further processed to remove potential outliers. Observations that situated >|3| standard deviations from the population mean based on a normal distribution were excluded. This still enabled using 99.7% of the original pixel data.

In general, heterogeneity can be used for describing the chemical or physical properties of a material. Chemical heterogeneity generally relates to composition, while physical heterogeneity is often related to particle size or shape distribution. For many materials heterogeneity is a mixture of the two. We described heterogeneity of the samples through the concept of constitutional heterogeneity (h_i_), originally developed within the theory of sampling to account for weight differences of different sub-samples^24^. However, we assumed constant mass and penetration depth across all pixels and expressed h_i_ as:

5 $${h}_{i}=N\sum _{i=1}^{N}\frac{{({c}_{i}-\bar{c})}^{2}}{{\bar{c}}^{2}}$$

where *c*_*i*_ denoted the carbon concentration in a single pixel (%, daf), $\bar{c}$ the average concentration of all pixels in a sample, i.e., predicted sample concentration (%, daf) and N the number of pixels within a sample. This definition provided a dimensionless heterogeneity metric corrected for the number of pixels between different samples that can be used for comparing different sample properties. In addition, the metric took into account increases in spatial detail as heterogeneity generally increases with an increasing number of image pixels.

**Figure 3:**
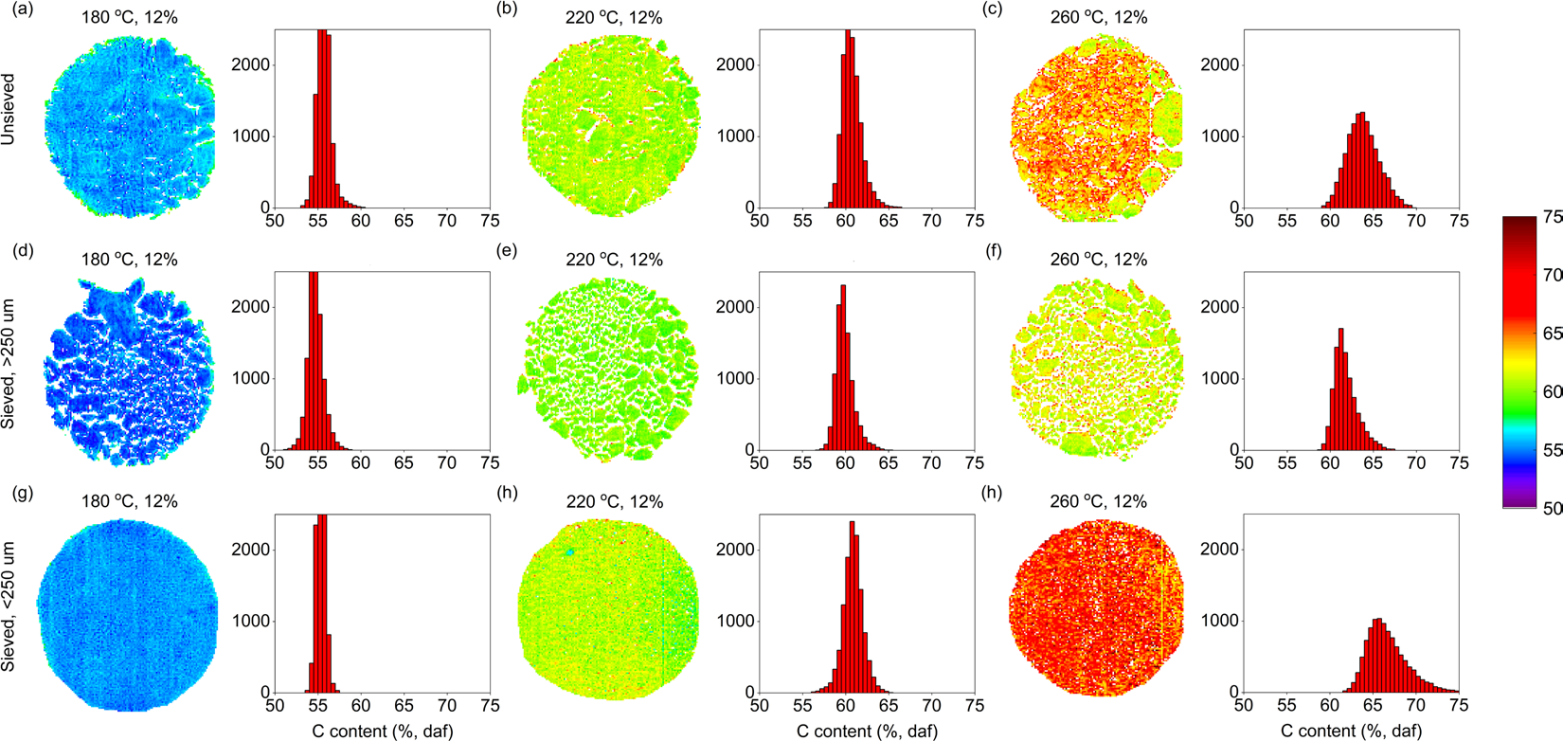
Examples of the predicted carbon contents (%, daf) of different samples. The histograms illustrate pixel count for different carbon contents. Sample labels show carbonization conditions given in Table 1. Determined heterogeneities, h_i_·10^4^: (**a**) 3.8, (**b**) 5.8, (**c**) 13, (**d**) 3.4, (**e**) 2.9, (**f**) 4.2, (**g**) 1.2, (**h**) 5.3 and (**i**) 14.

As illustrated in Fig. 3, the carbon contents and respective heterogeneity increased gradually with increasing carbonization temperature. Higher temperatures hence generated more variation in the carbon content of the samples. The distributions also seemed to be affected by the different size fractions. It is currently well known that temperature governs hydrothermal reactions and higher temperatures lead to increased decomposition of the lignocellulosic components normally present in biomass^25,26^. The raw calibration spectra indicated the loss of O-H, C-O and C-H bonds suggesting the decomposition of carbohydrates. The histograms in Fig. 3 give an indication of the progress of hydrothermal reactions. Higher temperatures suggested increased reaction rates especially for smaller particles. We have previously shown that the variation in pixel predictions decreased with the severity of carbonization after the char samples from different waste feeds were milled to obtain undistorted spectra^27^. However, a robust heterogeneity metric should be able to determine changes in heterogeneity without specific sample pretreatment.

**Figure 4:**
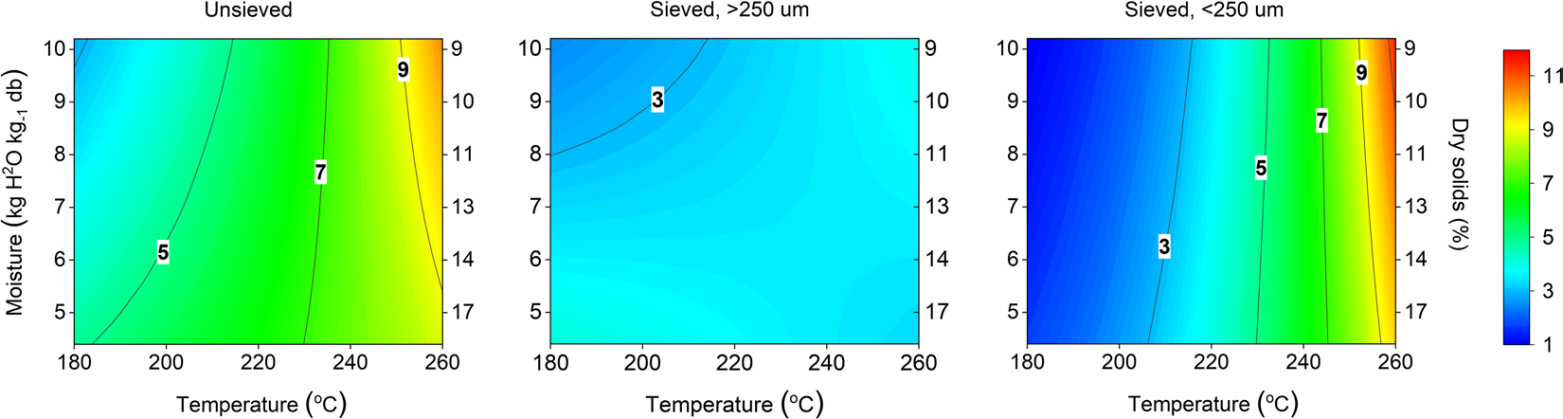
Response surfaces of predicted heterogeneity for different sample fractions based on carbonization conditions.

As the original samples were prepared based on an experimental design, the determined heterogeneities could also be described based on a regression model. This allows evaluating the effects of carbonization conditions on the heterogeneity metric and to illustrate changes through response surfaces. An interaction model based on log10 transformed response values explained 90% of data variation with no significant lack of fit. Different sample size fractions were described by two dummy variables. The model showed that both temperature and sample size fraction had a statistically significant effect (p < 0.001) on heterogeneity and also showed a significant interaction (p < 0.001). Moisture content was the least significant variable (p = 0.10) but showed a significant interaction with temperature (p = 0.01). The standard error of the model was 0.10 within a range of 1.16 log10 transformed heterogeneities (Table 2). As illustrated in Fig. 4, the model predictions confirmed that heterogeneity increased with carbonization temperature. The sieving procedure also significantly decreased heterogeneity in the larger particle size fraction. Particles smaller than 250 μm included a wider range of carbon contents that were nearly comparable to the original unsieved samples. As sample heterogeneity can be determined and modelled, it can also be controlled. This opens up doors for enhancing the quality of renewable carbon materials.

**Table 2:** Analysis of variance for the regression model on the effects of carbonization conditions on determined heterogeneities.

Source	Sum of squares	Degrees of freedom	Mean square	F-ratio	p-value
Total corrected	2.2	32			
Model	1.97	7	0.28	31	<0.01
Residual	0.23	25	9.1 10^−3^		
Lack of fit	0.16	19	8.6 10^−3^	0.79	0.68
Pure error	0.07	6	0.01		

The heterogeneity values were log10 transformed.

To the better of our knowledge, this is the first time that hyperspectral NIR imaging has been used to quantify the constitutional heterogeneity of a predicted analyte distribution. The concept of heterogeneity based on the theory of sampling has previously been discussed by Piqueras *et al*.^28^ in an imaging context. The authors used the standard deviations of predicted constituent distributions determined through multivariate curve resolution as constitutional heterogeneity estimates. In our work, their method did not provide robust heterogeneity estimates as the metric is highly sensitive to outlier removal. However, the authors made important observations on the differences between constitutional and distributional heterogeneity when applied to images. As constitutional heterogeneity describes the variation of independent pixel values around the mean, the spatial structure of the image is lost^28^. Thus heterogeneity is best interpreted by also considering the predicted concentration image (Fig. 3), which together provide information on both the constitutional and distributional heterogeneities. For renewable carbon materials, determination of heterogeneity enables developing methods to minimize variation and increase the quality of the carbon material for subsequent applications. This will help to develop renewable carbon materials for replacing respective fossil alternatives in a wide range of energy and environmental applications. This method can also be used for characterizing other materials that absorb in the NIR region.

## Conclusions

Heterogeneity affects material quality. Reliable quantification of heterogeneity has traditionally been a challenging task, as it requires rigorous sampling and analysis procedures. Spectral imaging can determine the spatial distribution of an analyte in sample, thus transforming each pixel into a sampling cell. With a large number of image pixels, the results can be evaluated using population statistics, which allows determining a robust heterogeneity metric for biological materials. Here we have shown that hyperspectral NIR imaging can be used for reliable determination of heterogeneity of biomass-derived carbon after multivariate image regression. Heterogeneity can also be modelled and controlled based on carbonization conditions. In the future, the variation and quality of carbon materials can potentially be monitored online within production systems. The concept of heterogeneity should however be extended beyond the imaged surface a material. Future work should also determine the effect of pixel size on reliable heterogeneity estimates and separate physical and chemical heterogeneities.
